# T-Box Transcription Factor 22 Is an Immune Microenvironment-Related Biomarker Associated With the *BRAF*^*V*600*E*^ Mutation in Papillary Thyroid Carcinoma

**DOI:** 10.3389/fcell.2020.590898

**Published:** 2020-12-17

**Authors:** Xubin Dong, Jingjing Song, Jing Hu, Cheng Zheng, Xiaohua Zhang, Haiguang Liu

**Affiliations:** ^1^Department of Thyroid and Breast Surgery, The First Affiliated Hospital of Wenzhou Medical University, Wenzhou, China; ^2^Department of Children’s Health Care, The Second Affiliated Hospital and Yuying Children’s Hospital of Wenzhou Medical University, Wenzhou, China; ^3^Department of Gastrointestinal Surgery, People’s Hospital of Yueqing, Wenzhou, China

**Keywords:** TBX22, papillary thyroid cancer, biomarker, tumor microenvironment, *BRAF*^*V*600*E*^, RAS

## Abstract

Papillary thyroid cancer (PTC) is the most common malignant disease in endocrine systems. T-box transcription factor 22 (TBX22) is a phylogenetically conserved family member that has not been widely characterized in cancers. In this study, we explored the potential clinical significance and biological functions of *TBX22* in PTC. Comprehensive analyses of *TBX22* were based on the public databases and our local qRT-PCR cohort. We observed that *TBX22* was significantly downregulated in PTC compared with normal tissues. *TBX22* was associated with several clinicopathological factors in PTC. Low *TBX22* expression correlated with *BRAF*^*V*600*E*^ and TERT mutation. Functional enrichment analysis revealed that cancer-related pathways and immune progress were closely associated with *TBX22* in PTC. In TBX22-low PTC, high immune infiltration levels with increased CD8^+^ T cells, natural killer, M1 macrophages, and T-regulatory cells were observed. *TBX22* was negatively correlated with the activity of different steps of the anticancer immunity cycle. Functionally, overexpression of *TBX22* inhibited the proliferation, invasion, and migration in PTC cells, while knocking down of *TBX22* showed the opposite effects. The present findings disclose that *TBX22*, as an immune microenvironment-related biomarker, could be an important tumor suppresser gene and might inform the management of PTC patients better.

## Introduction

According to the origin of cells, thyroid cancers are classified into follicular-derived and C-cell-derived. Follicular-derived thyroid cancers are subdivided into anaplastic, papillary (PTC), and follicular thyroid cancers (Landa et al., 2016). Over the past several decades, the rising incidence of PTC has been reported, making it an increasingly important health tissue in most populations of the world (James et al., 2018). Clinical and pathologic factors associated with a somewhat higher risk for PTC recurrence and cancer-related mortality include older age at diagnosis, size of the primary tumor, and the presence of soft-tissue invasion or distant metastases (Johnson et al., 1988; Asioli et al., 2010; Randolph et al., 2012). The prognosis is poorer in patients with specific subtypes of PTC, including tall cell and hobnail variants (Yoo et al., 2016). PTC patients had a favorable overall prognosis, and cancer-specific mortality in non-metastatic PTC patients was only 6% (Ghossein and Livolsi, 2008). However, existing treatments do not sufficiently improve the prognosis of more aggressive and lethal dedifferentiated PTCs.

Papillary thyroid cancer is MAPK-driven cancer with two mutually exclusive drivers, *BRAF*^*V*600*E*^ and mutated *RAS*, leading to distinct signaling consequences. Based on this, *BRAF*^*V*600*E*^-like (BVL) and RAS-like (RL) are two major PTC molecular subtypes that represent differential regulation of MAPK signaling pathways and thyroid differentiation (Agrawal et al., 2014). RL-PTCs enriched for follicular variant histology result in predominantly well-differentiated tumors, while BVL-PTCs enriched for classical and tall cell histology result in the less differentiated tumor (Ghossein et al., 2007).

The thyroid is the largest endocrine organ in the body and a common target of autoimmune disease. Immune checkpoint blockade like CTLA-4 and PD-1/PD-L1 inhibitors has fueled the field of tumor immunology in thyroid cancer, highlighting both the ability of tumor–immune system treatment and the uniqueness of tumor immunotherapy (Emens et al., 2017). The initiation and progression of the tumor depend on the interaction between the immune system, the TME, and individual tumor cells (Dong et al., 2020a,b). TME is a complex dynamic cell community composed of tumor epithelial cells and a variety of tumor-supporting cells, which represents an important contributor to immortal proliferation, cell invasion, and evasion of immune surveillance (Cheng et al., 2019). Previous studies uncovered that TIICs affect the response rate of immunotherapy, the efficacy of chemotherapy, and the prognosis of patients (Deschoolmeester et al., 2010). There are cancer-promoting and anticancer immune cells within the TIME. The predominance of procancer over anticancer immune cells may be related to cancer progression (Wu et al., 2017). Chen and Mellman (2013) demonstrated the seven steps of the cancer immunity cycle, which have become the basic framework of cancer immunotherapy research. Therefore, it is necessary to evaluate the immune properties of TME to identify the characteristics of novel predictive biomarkers and immune-related molecular in PTC.

T-box is named because this family shares a common DNA-binding domain (Sebé-Pedrós et al., 2013). The T-box genes constitute an ancient family of evolutionarily conserved transcription factors that are crucial to embryonic development by regulating gene expression (Papaioannou, 2014). The T-box gene family was widely studied in the field of developmental biology. Previous studies had demonstrated that mutations in different members of the T-box family result in developmental syndromes, characterized by organ deformity and body structure defects (Miller et al., 2010). In recent years, increasing studies reported that the T-box genes might also function in tumorigenesis and progression in certain cancers (Crawford et al., 2019; Krstic et al., 2019). As a member of the T-box gene family, T-box transcription factor 22 (*TBX22*) mutations caused a high risk of incidence in several diseases, including non-syndromic cleft palate, hypodontia, and ankyloglossia (Braybrook et al., 2001; Kantaputra et al., 2011). However, *TBX22* has so far received little attention in PTC studies.

Recent computational analyses of the RNA-seq profile of bulk tumors allow us to investigate TME in large cohorts. Here, in our study, we comprehensively investigated the expression pattern of *TBX22* and its association with the clinical and pathological parameters of PTC patients. Of note, we evaluated the clinical utility of *TBX22* by correlative analyses with PTC key molecular features, such as *BRAF*^*V*600*E*^, *RAS*, and *TERT* mutation. Based on functional enrichment analysis, we explored the link between *TBX22* and tumor immunity-associated molecular features. Therefore, we performed a series of experiments to demonstrate the effect of overexpression and knocking down *TBX22* on PTC cell lines. Our findings were largely consistent in public cohorts and our local PTC profile.

## Materials and Methods

### Bioinformatics

This study applied multiple public PTC cohorts. For The Cancer Genome Atlas (TCGA) dataset, RNA-seq and clinical profiles of human thyroid cancer patients were obtained from the TCGA data portal (Agrawal et al., 2014). TCGA RNA-seq profile contained 568 samples, comprising 59 matched normal samples, 501 PTC samples, and eight metastatic thyroid carcinoma samples. Whole-transcriptome sequencing data were aligned using RSEM expression level in transcripts per million. The definition and outcome of DFI and PFI were extracted from the TCGA-Clinical Data Resource (Liu et al., 2018). Mutation status was obtained from Mutation Annotation Format files (derived from VarScan2) from the Genomic Data Commons portal. TMB was calculated by the sum of the number of non-silent mutations in the TCGA samples. MSI status (MANTIS score) for the TCGA cohort was obtained from a previous publication (Bonneville et al., 2017). Expressional data and corresponding clinical profiles of GSE33630 (Dom et al., 2012), GSE60542 (Tarabichi et al., 2015), and GSE35570 (Yu et al., 2008; Handkiewicz-Junak et al., 2016) datasets were obtained from the GEO database.

Correlation analysis was assessed by the function “cor.test” in R. Genes which had Spearman correlation value >0.4 or <−0.4, *p* < 0.001 with *TBX22* were selected. GO and KEGG analyses were performed using “enrichGo” and “enrichKEGG” functions in the “clusterprofiler” package, respectively. “ggplot2” and “pheatmap” packages were used for visualization. The GSEA was performed using the hallmark collections from the Molecular Signature Database (Subramanian et al., 2005). FDR-adjusted *q*–value <0.25 was set as the cutoff to identify significantly enriched terms.

The ESTIMATE algorithm was used to quantify the immune and stromal proportions in the PTC microenvironment (Yoshihara et al., 2013). For specific TIIC infiltration analyses, quanTIseq and ImmuCellAI algorithms were used to estimate the relative proportions of immune cell types in TME. ImmuCellAI is a gene-expression-based pipeline for the quantification of the tumor immune contexture from human RNA-seq profiles (Miao et al., 2020). quanTIseq is a deconvolution-based algorithm, providing “absolute score” representing cell fractions in TME (Finotello et al., 2019). The TIP pipeline was applied for cancer immunity cycle profiling (Xu et al., 2018). This method used an algorithm based on ssGSEA to evaluate the relative activity of the seven steps of the immune cycle, which is a process by which the immune system recognizes and kills cancer cells in a large number of tumor samples.

### Patients and Thyroid Tissue Samples

Seventy-nine pairs of PTC and corresponding normal thyroid tissues were obtained from the Department of Thyroid and Breast Surgery, The First Affiliated Hospital of Wenzhou Medical University. Furthermore, all surgical specimens were independently confirmed by two experienced pathologists, and the results showed that 79 pairs of surgical specimens all met the diagnosis of PTC and normal thyroid tissues, respectively. The collected fresh tissues were immediately snap-frozen in liquid nitrogen and stored at −80°C until further RNA was detected. *BRAF*^*V*600*E*^ mutation detection was performed on the extracted DNA from preoperative fine-needle aspiration cells after cytologic evaluation. Briefly, each real-time PCR mixture contained 5 μl of extracted DNA and other reagents available in the detection kit ADx-Amplification Refractory Mutation System (AmoyDx, China). Clinical and pathological features of patients in the TCGA and WMU-THCA cohorts are presented in Tables 1, 2, respectively.

**TABLE 1:** Baseline of patients in the TCGA cohort.

	**Level**	**Overall**
*n*		501
Age (median [IQR])		46.00 [35.00, 58.00]
Race (%)	American Indian or Alaska native	1 (0.2)
	Asian	52 (12.7)
	Black or African American	26 (6.3)
	White	331 (80.7)
Gender (%)	Female	366 (73.1)
	Male	135 (26.9)
Pathologic stage (%)	Stage I	282 (56.5)
	Stage II	51 (10.2)
	Stage III	111 (22.2)
	Stage IV	55 (11.0)
Pathology T stage (%)	T1	141 (28.1)
	T2	166 (33.1)
	T3	169 (33.7)
	T4	23 (4.6)
Pathology N stage (%)	N0	226 (45.1)
	N1	226 (45.1)
Pathology M stage (%)	M0	279 (55.8)
	M1	9 (1.8)
Radiation therapy (%)	No	180 (37.1)
	Yes	305 (62.9)
Histological type (%)	Others, specify	7 (1.4)
	Classical/usual	357 (71.3)
	Follicular	102 (20.4)
	Tall cell	35 (7.0)
Radiation exposure (%)	No	421 (96.1)
	Yes	17 (3.9)
Extrathyroidal extension (%)	Minimal (t3)	133 (27.5)
	Moderate/advanced (t4a)	18 (3.7)
	None	331 (68.5)
	Very advanced (t4b)	1 (0.2)
Residual tumor (%)	R0	383 (81.7)
	R1	52 (11.1)
	R2	4 (0.9)
Multifocality (%)	Multifocal	227 (46.2)
	Unifocal	264 (53.8)

**TABLE 2:** Baseline of patients in the WMU-THCA cohort.

	**Level**	**Overall**
*n*		79
Age (median [IQR])		43.00 [33.00, 52.00]
Gender (%)	Female	59 (74.7)
	Male	20 (25.3)
Pathology T stage (%)	T1	60 (75.9)
	T2	13 (16.5)
	T3	6 (7.6)
Extrathyroidal extension (%)	None	65 (82.3)
	Yes	14 (17.7)
Pathology N stage (%)	N0	31 (39.2)
	N1	48 (60.8)
*BRAF*^*V*600*E*^ (%)	Negative	11 (13.9)
	Positive	48 (60.8)
AJCC7 stage (%)	Stage I	30 (38.0)
	Stage II	46 (58.2)
	Stage III	3 (3.8)
Envelop invasion (%)	No	24 (30.3)
	Yes	8 (10.1)
Multifocality (%)	Multifocal	33 (41.8)
	Unifocal	46 (58.2)

### qRT-PCR

Total RNA was isolated from patient tissue and thyroid cancer cell lines by TRIzol reagent (Invitrogen, United States). All RNA samples were temporarily stored at −80°C. The isolated RNA was measured at 260/280 nm to ensure the reliability of RNA quality and quantity. The range of 260/280 nm is between 1.72 and 1.95. The ReverTra Ace qPCR RT Kit (Toyobo, Japan) was used for RNA reverse transcription. Real-time PCR was run and analyzed using the 7500 Fast quantitative PCR System (Applied Biosystems, United States). The relative expression of *TBX22* mRNA was presented using the method of 2^–ΔΔ*CT*^ with the endogenous control *GAPDH* to normalize the data. The primer sequences used were as follows: *TBX22* forward primer, 5′-CCUUUGAACUCCUUACUUUTT-3′; *TBX22* reverse primer, 5′-AAAGUAAGGAGUUCAAAGGTT-3′; *GAPDH* forward primer, 5′-GTCTCCTCTGACTTCAACAGCG-3′; *GAPDH* reverse primer, 5′-ACCACCCTGTTGCTGTAGCCAA-3′.

### Cell Cultures and Transient Transfection With Plasmids or siRNAs

BCPAP, TPC-1, and KTC-1 cells were cultured in Roswell Park Memorial Institute 1640 medium (Gibco, United States) supplemented with 10% FBS (PAN Biotech, Germany). DMEM (Gibco, United States) supplemented with 10% FBS was used for growing FTC and HTori-3 cells. These cells were incubated in a humidified incubator at 37°C with 5% CO_2_. Cells used in the present study were all obtained from Shanghai Cell Biology, Institute of the Chinese Academy of Sciences (Shanghai, China). Employed cell lines have different genotypes, as shown in Table 3.

**TABLE 3:** Genotype of the employed PTC cell lines.

**Cell lines**	**Category**	***BRAF***	***NRAS***	***HRAS***	***KRAS***	***TERT***	***RET*/*PTC1***	**Resource**
BCPAP	PTC cell lines	GTG- > GAG V600E*	wt	wt	wt	c.1-124C > T (c.228C > T)^†^	wt	Meireles et al., 2007; Landa et al., 2019
TPC-1	PTC cell lines	wt	wt	wt	wt	c.1-124C > T (c.228C > T)^†^	+	Meireles et al., 2007; Landa et al., 2019
KTC-1	PTC cell lines	GTG- > GAG V600E*	wt	wt	wt	c.1-146C > T (c.250C > T)^†^	wt	Landa et al., 2013, 2019

wt, wild-type genotype; +, presence of the gene rearrangement. ^†^TERT promoter mutations. *Heterozygous mutation.

For *TBX22* overexpression, the structure of vectors contained the full length of *TBX22* and was constructed into CMV-MCS-EGFP-SV40-Neomycin. pcDNA3.1-*TBX22* and its empty vector were purchased from GeneChem (Shanghai, China) according to the manufacturer’s recommendations. Transient siRNA transfection for *TBX22* was obtained from RiboBio (Guangzhou, China) for cell interference. Based on the manufacturer’s protocol, Lipofectamine RNAiMAX transfection reagent (Thermo Fisher Scientific, United States) was mixed with siRNA to transfect cancer cell lines. The negative control (si-NC) was not homologous to any human genomic sequence tracks. The siRNA sequences are listed as follows: si-TBX22: sense: 5′-CCUUUGAACUCCUUACUUUTT-3′, antisense: 5′-AAAGUAAGGAGUUCAAAGGTT-3′.

### Cellular Proliferation, Migration, and Invasion Assay

To determine the ability of cell proliferation, the Cell Counting Kit-8 (CCK-8, Beyotime, China) was employed in this study. Forty-eight hours after siRNA transfection, 1,500 cells were seeded into each well of a 96-well plate with 100 μl medium supplemented with 10% FBS. At every indicated time point, the medium was exchanged by 100 μl medium with CCK-8 (91 μl medium and 9 μl CCK-8), and the cells were incubated in 5% CO_2_, 37°C for 3 h. The OD at a wavelength of 450 nm in each well was recorded on a microplate reader (SpectraMax Plus 384, Molecular Devices Corporation, CA, United States). For the colony formation assay, about 1,000 transfected cancer cells were added to each well in a six-well plate and incubated for 10–14 days until colonies were formed. The plates were then gently washed with PBS and stained with crystal violet. Colony areas were determined by the ColonyArea plugin (Guzman et al., 2014) in ImageJ software. Transwell plates (Corning, NY, United States) were used for the migration assay. Cells (4 × 10^4^ cells/chamber) were seeded onto the upper chamber, and the growth medium containing 10% FBS was added to the bottom chamber. After 24 h and 37°C incubation, non-migrated cells on the upper chamber were carefully removed with a cotton swab. Cells migrating through the filter chamber were fixed with methanol and stained with 0.1% crystal violet. For the invasion assay, an identical protocol was followed, except that the chamber is Matrigel invasion chamber (Corning Incorporated). Migrated or invaded cells were imaged in a 10× magnification microscope in five random fields for each well and quantified by ImageJ software.

### Statistical Analysis

Mann–Whitney test or Wilcoxon signed-rank test was used in the two-group analysis. Comparisons between different groups were conducted by Kruskal–Wallis one-way ANOVA. Correlation analyses between two variables were calculated using Spearman Rho. These variables included gene expression levels, TME signature scores, TMB, and MSI scores. The ROC curve analysis estimated the biomarker value of *TBX22*. and the Kaplan–Meier curves and log-rank tests were used to evaluate differences in prognosis. The optimal *TBX22* cutpoints for Kaplan–Meier curves were calculated using the “res.cut” function. Patients with full clinical information were selected for the Cox regression analysis, and we removed patients with a follow-up period of fewer than 30 days. Uni- and multivariable Cox regression models were built using the clinical and pathological parameters along with the respective *TBX22* expression data by the “coxph” function in the “survival” package. R 3.5.2 and GraphPad Prism 8.2.0 were utilized in this study.

## Results

### The Expression Level of *TBX22* in PTC

**FIGURE 1:**
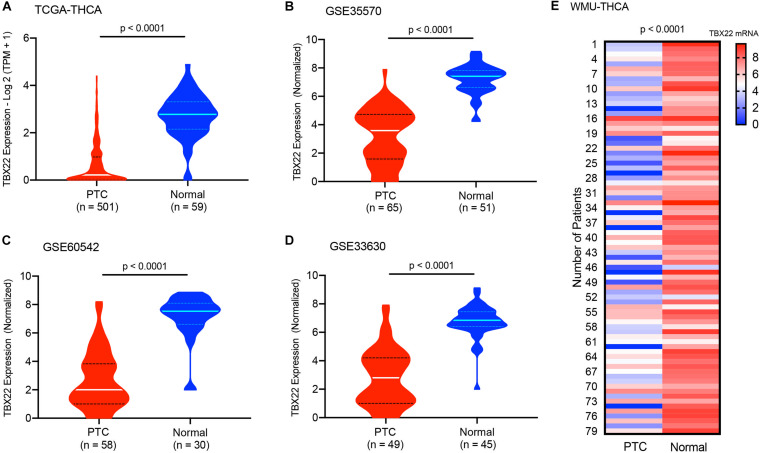
T-box transcription factor 22 (*TBX22*) levels in normal and malignant thyroid tissues. The *TBX22* transcript levels in papillary thyroid cancer (PTC) and adjacent normal thyroid tissues from the **(A)** TCGA-THCA, **(B)** GSE35570, **(C)** GSE60542, and **(D)** GSE33630 datasets are presented as violin plots. **(E)** *TBX22* expression in local THCA tissues compared with the paired normal thyroid samples by qRT-PCR. Statistical methods were as follows: **(A–D)** Wilcoxon rank-sum test; **(E)** Wilcoxon signed-rank test.

For PTC patients (TCGA cohort), *TBX22* was significantly lower in PTC than in the corresponding normal thyroid tissue (Figure 1A, *p* < 0.0001), which was also compatible in the GSE33630, GSE60542, and GSE5364 cohorts (Figures 1B–D, all *p* < 0.0001). To validate the transcription level of *TBX22* in our local PTC patients, we performed qRT-PCR of 79 pairs of tumor samples and adjacent normal thyroid tissues, and *TBX22* was downregulated in PTC tissues compared with that in adjacent normal tissues (Figure 1E, *p* < 0.0001). These results demonstrated that *TBX22* was downregulated in PTC.

### The Expression of *TBX22* Was Negatively Correlated With the Development of PTC

**FIGURE 2:**
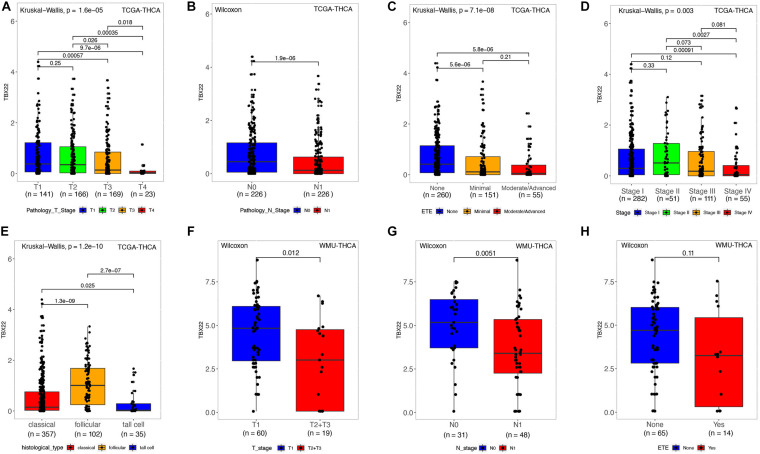
*TBX22* expression and correlation with clinicopathological characteristics in PTC patients. **(A–E)** In the TCGA-THCA dataset, *TBX22* expression in the different status of tumor size, lymph node metastasis, extrathyroidal extension, disease stages, and histological types. **(F–H)** In the local cohort, *TBX22* expression in the different status of tumor size, lymph node metastasis, and extrathyroidal extension. **(A,C–E)** Kruskal–Wallis test; **(B,F–H)** Wilcoxon rank-sum test.

Next, we analyzed the relationship between mRNA expression of *TBX22* and the PTC clinicopathological parameters in the TCGA database and the local WMU cohort. In the TCGA cohort, the mRNA level of *TBX22* in PTC patients was significantly associated with tumor size (Figure 2A, *p* = 1.6e-05), nodal metastasis (Figure 2B, *p* = 1.9e-06), extrathyroidal extension (Figure 2C, *p* = 7.1e-08), and disease stage (Figure 2D, *p* = 0.003). In addition, *TBX22* level was significantly higher in the follicular variant subtype compared with the tall cell variant (Figure 2E, *p* = 1.6e-05) and classical subtypes (Figure 2E, *p* = 1.3e-09). Our further analysis of PTC profiles of the local cohort validated that the expression of *TBX22* was significantly associated with tumor size (Figure 2F, *p* = 0.012) and LNM (Figure 2G, *p* = 0.0051). We also observed that *TBX22* had a trend in distinguishing between PTC patients with and without extrathyroidal extension (Figure 2H, *p* = 0.11).

### Association Between *TBX22* and Molecular Characteristics

**FIGURE 3:**
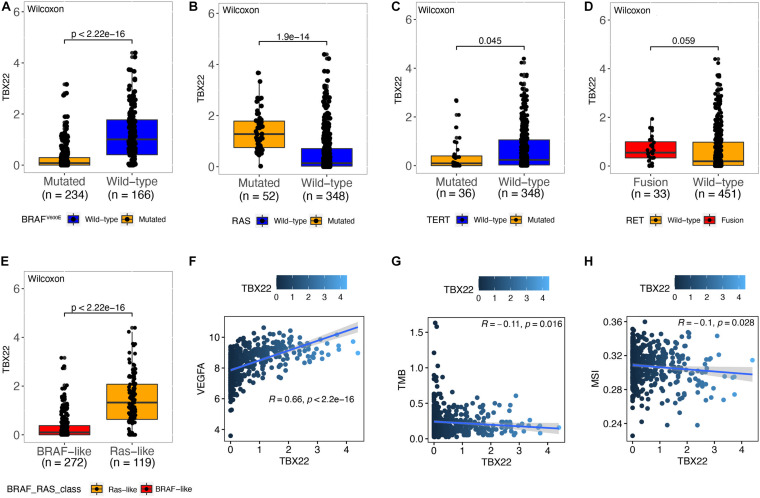
The relationship between *TBX22* expression and molecular features of PTC. **(A–E)** Distribution of the *TBX22* expression in PTC patients stratified by **(A)** *BRAF*^*V*600*E*^ status, **(B)** *RAS* status, **(C)** *TERT* status, **(D)** *RET* status, and **(E)** BRAF-RAS-class. **(F–H)** Spearman correlations between *TBX22* and **(F)** *VEGFA* expression, **(G)** TMB, and **(H)** MSI score in PTC patients.

Papillary thyroid cancer can be classified according to 71 gene expression signatures as BVL and RL (Cancer Genome Atlas Research Network, 2014). Thus, we divided patients according to their driver mutation status and found that *TBX22* expression was significantly lower in the *BRAF*^*V*600*E*^ (Figure 3A, *p* < 0.0001) and *TERT* mutation groups (Figure 3C, *p* = 0.045). However, the expression of *TBX22* was higher in the *RAS* wild-type group (Figure 3B, *p* < 0.0001) than in their respective counterpart groups. Besides, *TBX22* showed a trend in distinguishing between the *RET* fusion group and the *RET* wild-type group (Figure 3D, *p* = 0.059). *TBX22* expression was significantly lower in the BVL group than in the RL group (Figure 3E, *p* < 0.0001). In addition, *TBX22* showed a strong positive correlation with *VEGFA* (Figure 3F, *R* = 0.66, *p* < 0.0001). However, *TBX22* was weakly correlated with scores of TMB (Figure 3G, *R* = -0.11, *p* = 0.016) and MSI (Figure 3H, *R* = −0.1, *p* = 0.028).

### *TBX22* Could Serve as a Robust Biomarker in PTC Patients

**FIGURE 4:**
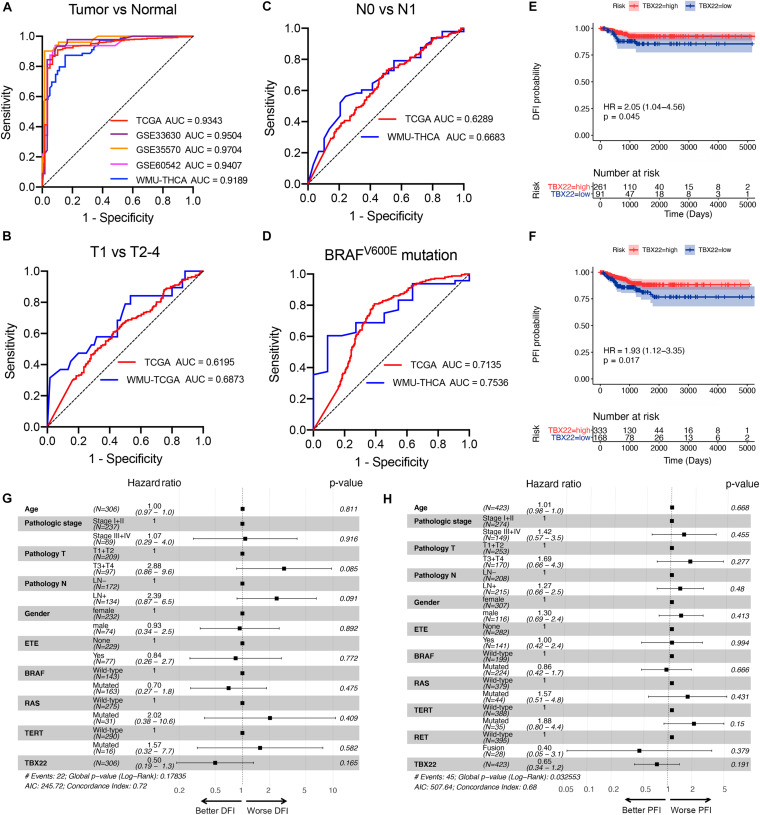
*TBX22* is a potential predictive biomarker in PTC. **(A)** ROC curves showed the diagnostic values of *TBX22* in public PTC datasets and our local cohort. **(B–D)** ROC curve analysis shows predictive efficiency of *TBX22* in **(B)** T stage, **(C)** LNM status, and **(D)** *BRAF*^*V*600*E*^ status. **(E,F)** Comparison of Kaplan–Meier curves of patients with high and low *TBX22* expression along with log-rank test *p*-values and hazard ratios (HR) with confidence intervals is shown for **(E)** DFI and **(F)** PFI in the TCGA cohort. DFI, disease-free interval. PFI, progression-free interval. **(G,H)** Multivariable Cox regression analyses of DFI **(G)** and PFI **(H)** in PTC patients.

Based on these results, we hypothesized that *TBX22* could serve as a biomarker for PTC and ROC curve analysis demonstrated that *TBX22* was diagnostically significant with an AUC value of 0.9343, 0.9504, 0.9704, 0.9407, and 0.9189 in the TCGA, GSE33630, GSE35570, GSE60542, and our validated local cohorts, respectively (Figure 4A). *TBX22* also had predicted value for the T stage in TCGA cohort (AUC = 0.6195) and our local cohort (AUC = 0.6873, Figure 4B). For LNM, the AUC was 0.6289 in TCGA and 0.6683 in our validated cohort (Figure 4C). Importantly, the AUC for distinguishing *BRAF*^*V*600*E*^ (mutated vs. wild type) was 0.7135 in TCGA and 0.7536 in our local validated cohort (Figure 4D). Our results suggested that *TBX22* could be exploited as a reliable predictive biomarker for PTC patients.

We hypothesized that *TBX22* could be a prognostic biomarker for PTC patients. In the survival analysis, *TBX22* expression alone is significantly associated with better DFI (Figure 4E, HR: 2.05; 95% CI: 1.04–4.56; *p* = 0.045) and PFI (Figure 4F, HR: 1.93; 95% CI: 1.12–3.35; *p* = 0.017) in PTC patients. Using univariate Cox regression analyses, we identified disease stage, T stage, extrathyroidal extension, LNM, and *TBX22* were significantly associated with DFI and PFI (Supplementary Table 1). However, we did not find independent prognostic factors in multivariate Cox regression analyses (Figures 4G,H).

### Predicted Functions and Pathways of *TBX22* in PTC

**FIGURE 5:**
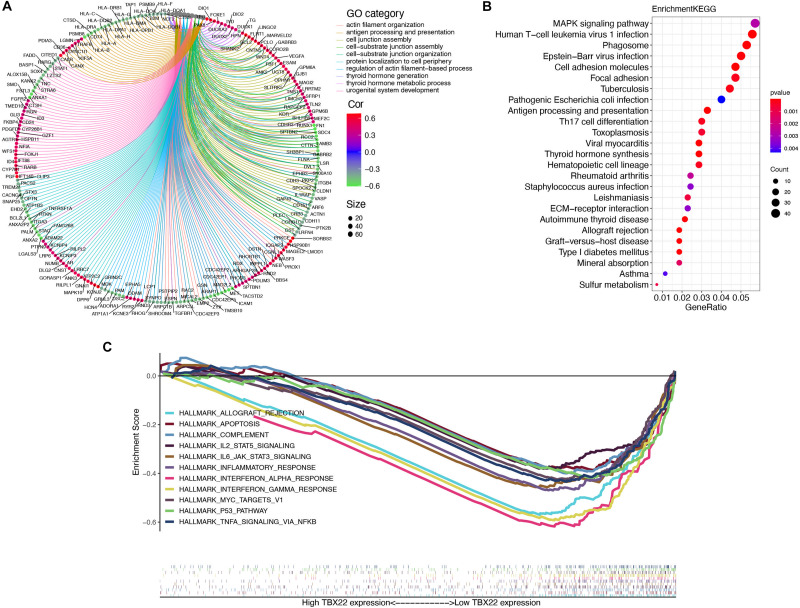
Organization of *TBX22* co-expressed genes and functional enrichment analysis. **(A,B)** Gene ontology **(A)** and KEGG pathways **(B)** were significantly correlated with *TBX22* expression of thyroid hormone metabolism, immune activation, and MAPK signaling pathways. The top *TBX22* co-expressed genes are shown (ranked by Spearman correlation value). **(C)** GSEA analyses displayed key pathways enriched in low (down) *TBX22* expression subset. The complete lists are given in Supplementary Tables 2–5.

In a specific biological environment, genes interact with each other and drive the underlying pathway and molecular function (Jia et al., 2016). Based on this, we utilized the TCGA dataset to perform co-expression analysis to elucidate the most relevant genes with *TBX22*. We further selected the genes with strong co-expression correlation with *TBX22* (Spearman correlation value >0.4 or <−0.4, *p* < 0.0001) for GO and KEGG analyses (Figures 5A,B and Supplementary Tables 2–4). GO enrichment and KEGG enrichment analyses unanimously suggested that *TBX22* is prevailingly correlated with thyroid hormone metabolism, immune activation, and MAPK signaling pathways. Consistently, GSEA *via* the hallmarks database revealed that the low *TBX22* subset was prevailingly enriched in various tumorigenesis pathways and immune-activating processes (Figure 5C and Supplementary Table 5). These well-reproductive results revealed that *TBX22* might have intrinsic roles in tumor immunity and PTC progression.

### *TBX22* Expression Correlates With TIICs and Cancer Immunity Cycle in TIME

**FIGURE 6:**
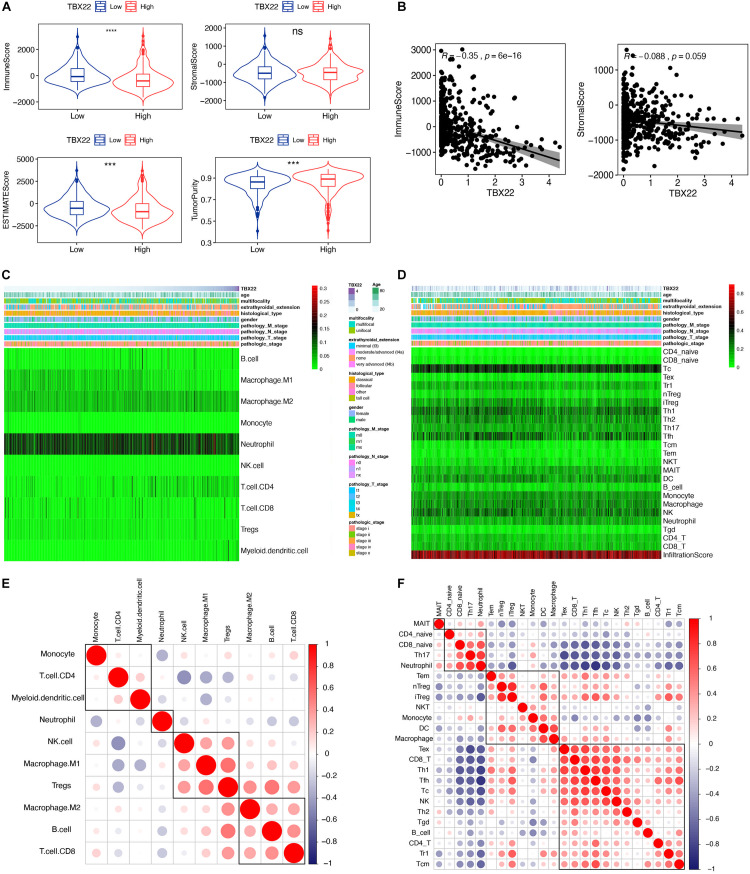
*TBX22* is related to tumor immune microenvironment phenotype. **(A)** Comparison of the immune score, stromal score, microenvironment score, and tumor purity between the TBX22^*high*^ and TBX22^*low*^ groups. The *p*-value, testing the group difference, was calculated with the two-sided Wilcoxon rank-sum test. **(B)** Spearman correlations between *TBX22* expression and immune and stromal scores. **(C,D)** Heatmap of the TIIC proportions in the PTC microenvironment quantified by **(C)** quanTIseq and **(D)** ImmuCellAI. **(E,F)** Spearman correlation matrix of the different TIIC proportions in the PTC microenvironment quantified by **(E)** quanTIseq and **(F)** ImmuCellAI. ***p < 0.001, ****p < 0.0001.

Previous studies have revealed that TIICs are extensively distributed in TME of PTC and influence the different stages of tumorigenesis and development. Stromal score and immune score represent the percentage of stromal cells and immune cells in the TME, respectively (Mao et al., 2013). TBX22^*high*^ and TBX22^*low*^ groups were divided based on the median value of the *TBX22* expression in the TCGA profiles. We adopted the ESTIMATE algorithm to evaluate the immune microenvironment and stromal microenvironment in the PTC samples. In the TBX22^*low*^ group, the immune score and TME score were higher, whereas tumor purity was lower compared with that in the TBX22^*high*^ group (Figure 6A). However, no significant association between stromal scores and *TBX22* expression was found (Figure 6B). In general, these analyses revealed that the expression of *TBX22* was negatively correlated with the immune scores in PTC. To explore the correlation of *TBX22* expression with the immune microenvironment, we inferred the abundance of TIICs by establishing two different algorithms, quanTIseq and ImmuCellAI. The landscapes of TIICs and clinical–pathological features are shown in Figures 6C,D. The hierarchical clustering of the whole-sample RNA-seq profile classified immune cells into four subgroups. Moreover, the proportions of TIICs were weakly to highly correlated (Figures 6E,F).

**FIGURE 7:**
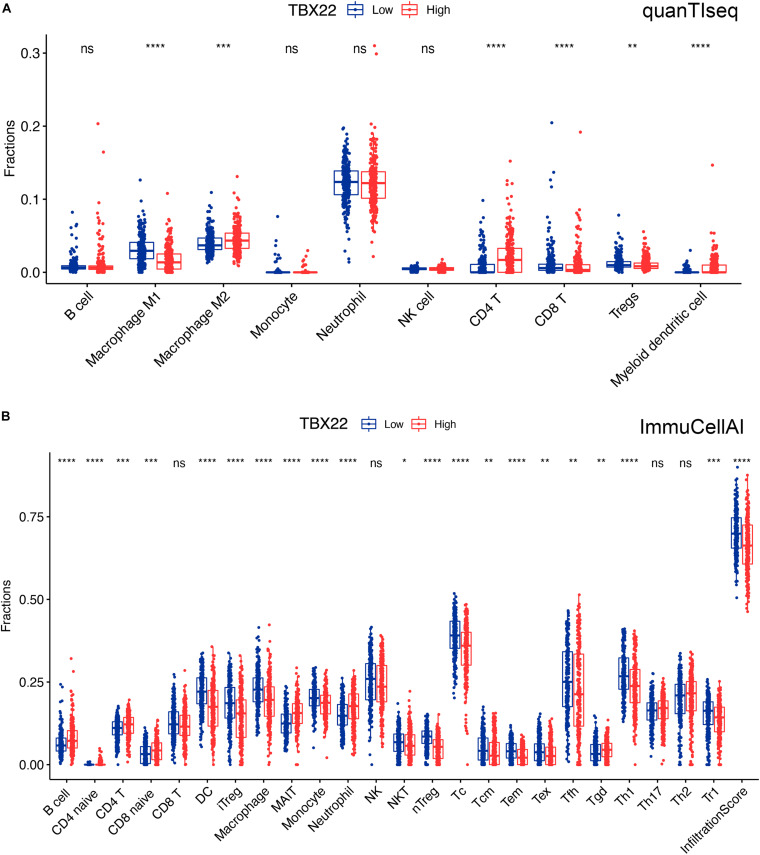
Boxplots of immune cells in TBX22^*high*^ and TBX22^*low*^ PTC in TCGA cohorts. Comparison of the immune infiltrated cell components quantified by **(A)** quanTIseq and **(B)** ImmuCellAI in the TBX22^*high*^ and TBX22^*low*^ groups. *p < 0.05, **p < 0.01, ***p < 0.001, ****p < 0.0001. The scattered dots represent all score values, and the thick line represents the median value within each group. The bottom and top of the boxes are the 25th and 75th percentiles (interquartile range). The difference between TBX22^*high*^ and TBX22^*low*^ groups was compared through the two-sided Wilcoxon rank-sum test.

We tried to determine whether the TIME was altered among patients with different levels of *TBX22* (Figures 7A,B). TBX22^*low*^ PTC was associated with significantly higher anticancer M1 macrophages and significantly lower procancer M2 macrophages; anticancer Th1 cells were higher in TBX22^*low*^ PTC, while procancer Th2 cells did not have a significant difference between different *TBX22* states. The levels of anticancer CD8^+^ T cells and procancer regulatory T cells were both elevated in TBX22^*low*^ PTC. At the same time, TBX22^*low*^ PTC had a higher number of anticancer T-follicular helper cells. NK cells did not have a significant difference between different *TBX22* states. Strikingly, CD4^+^ T cells were also lower in the TBX22^*low*^ PTC. ImmuCellAI infiltration score, an assessment of universal immune cytolytic activity, was also higher in the TBX22^*low*^ group (Figure 7B).

**FIGURE 8:**
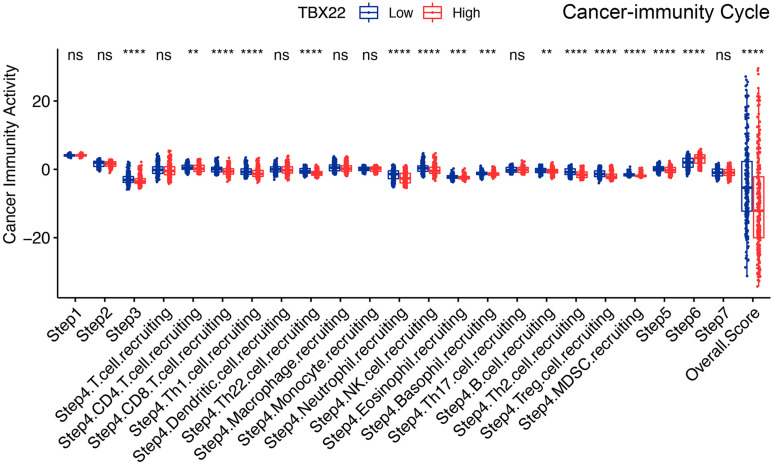
Comparison of the activity of the cancer immunity cycle between the TBX22^*high*^ and TBX22^*low*^ groups. **p < 0.01, ***p < 0.001, ****p < 0.0001. The scattered dots represent all score values, and the thick line represents the median value within each group. The bottom and top of the boxes are the 25th and 75th percentiles (interquartile range). The difference between TBX22^*high*^ and TBX22^*low*^ groups was compared through the two-sided Wilcoxon rank-sum test.

The cancer immunity cycle triggers a series of gradual events and enables the anticancer immune response to effectively kill cancer cells (Chen and Mellman, 2013). As shown in Figure 8, after releasing cancer cell antigen and presenting cancer antigen, effector T-cell responses against the cancer-specific antigens were primed and activated (step 1 to step 3). TBX22^*low*^ PTC had higher immune activity in step 3 compared with TBX22^*low*^. We observed that the TBX22^*low*^ PTC had higher anticancer immunity scores in the trafficking of T cells to tumors (step 4) and infiltration of T cells into tumors (step 5). TBX22^*low*^ PTC had a significantly higher level of recruiting CD8^+^ T cells, Th1, Th22, neutrophil, natural killer, eosinophil, basophil, B cells, Th2, and MDSCs. Interestingly, an elevated level of recognition of cancer cells by T cells (step 6) was observed in TBX22^*high*^ PTC. In summary, TBX22^*low*^ state may be associated with active anticancer immune response, and *TBX22* had a negative role in the cancer immunity cycle.

### Dysregulation of *TBX22* Affects Thyroid Cancer Cell Proliferation, Migration, and Invasion

**FIGURE 9:**
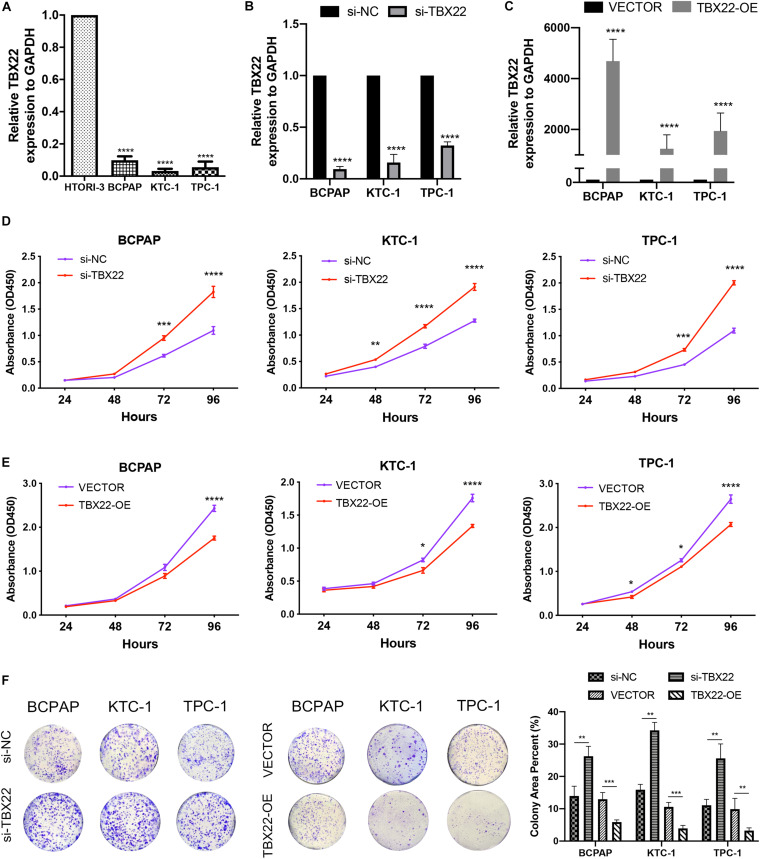
*TBX22* was downregulated in PTC cell lines and attenuated the capability of proliferation. **(A)** Relative *TBX22* mRNA expression levels were significantly lower in thyroid cancer cell lines (BCPAP, TPC-1, and KTC-1) compared with the normal thyroid epithelial cell line HTori-3. **(B)** Transcriptional level of *TBX22* in si-NC and si-TBX22 transfected PTC cells *via* qRT-PCR. **(C)** Transcriptional level of *TBX22* in VECTOR and TBX22-OE group *via* qRT-PCR. **(D,E)** CCK-8 assays determined the cell viability of **(D)** loss-of-TBX22 and **(E)** gain-of-TBX22 PTC cells. **(F)** Colony formation assays were used to determine the cell colony formation ability of loss-of-TBX22 and gain-of-TBX22 PTC cells. Student’s *t*-test was used in comparison with the si-NC or VECTOR groups. *p < 0.05, **p < 0.01, ***p < 0.001, ****p < 0.0001 in data analysis, and all assays were repeated at least three times.

To complement the above bioinformatic analyses, we verified the expression of *TBX22* in PTC cell lines. Compared with the normal thyroid cell lines (HTori-3), the *TBX22* level was significantly lower in PTC cell lines (BCPAP, TPC-1, and KTC-1) (Figure 9A). Next, we sought to elucidate the functional effects of *TBX22* dysregulation through studies *in vitro* on PTC cell lines.

We had found that *TBX22* was associated with T stage and disease stage in PTC patients (Figures 2A,D,F). To determine the function of *TBX22* in PTC tumorigenesis and progression, we knocked down *TBX22* in PTC cell lines using *TBX22*-targeting siRNA (si-TBX22). In contrast, we performed gain-of-function studies and generated control (VECTOR) and TBX22-overexpressing (TBX22-OE) PTC cell lines. The silenced (Figure 9B) and overexpressed (Figure 9C) efficiency of *TBX22* were highly effective at the mRNA level evaluated by qRT-PCR. Remarkably, si-TBX22 cells showed increased cell proliferation *in vitro* compared with si-NC cells (Figure 9D). On the other hand, TBX22-OE cells showed decreased cell proliferation *in vitro* compared with VECTOR cells (Figure 9E). In further colony formation assays, the si-TBX22 cells also showed increased colonies compared with si-NC cells, whereas TBX22-OE had decreased colonies compared with VECTOR cells (Figure 9F).

**FIGURE 10:**
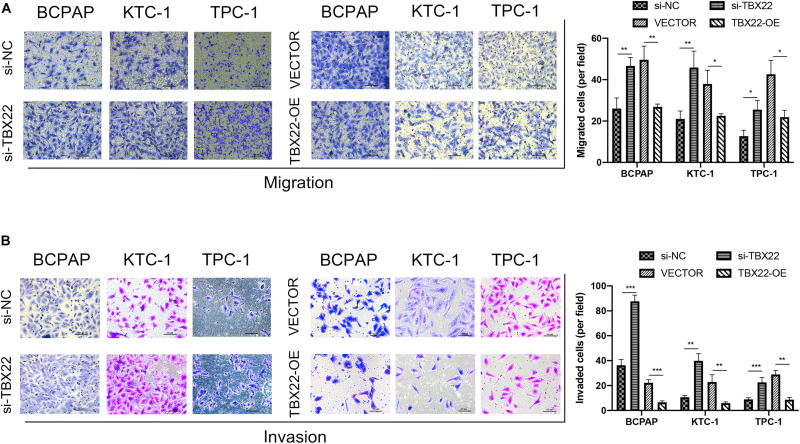
*TBX22* inhibited the migration and invasion of PTC cell lines. **(A,B)** Loss-of-function and gain-of-function assays indicated that *TBX22* reduced the **(A)** migration and **(B)** invasion abilities of PTC cell lines. *p < 0.05, **p < 0.01, ***p < 0.001 in comparison with the si-NC or VECTOR groups using Student’s *t*-test, and all assays were repeated at least three times.

We also had found that the expression of *TBX22* was associated with LNM and ETE in PTC patients (Figures 2B,C,G). To evaluate the impact of dysregulated *TBX22* on metastasis, we performed the assays of cell migration and invasion. si-TBX22 cells effectively increased migratory ability compared with those in the si-NC group, whereas TBX22-OE cells attenuated migratory ability compared with those in the VECTOR group (Figure 10A). Similar results were observed in invasion assays (Figure 10B). These data showed that *TBX22* might regulate the proliferation, migration, and invasion capacities of the PTC cells.

## Discussion

With the treatment of surgery and hormone replacement treatment, most PTC patients have a favorable prognosis. However, radiotherapy and chemotherapy are the only options for the treatment of undifferentiated, recurrent, and metastatic disease, with a median survival of less than 6 months (Mould et al., 2017; Tirrò et al., 2019). Na and Choi (2018) illustrated the close association between immune TME and immunotherapeutic implications in PTC. Thus, it is necessary to reveal the effective molecular mechanism and identify immune-related biomarkers of PTC to guide a more optimized treatment strategy. Recently, accumulating studies show that certain T-box genes also play an important role in cancer progression. Upregulated TBX2 and TBX3 promoted tumorigenesis and progression of different types of cancer (Durbin et al., 2018; Crawford et al., 2019; Krstic et al., 2019). In contrast, a recent study revealed that TBX1 functions as a tumor suppressor in PTC (Wang et al., 2019). As a member of the T-box transcription factor gene family, *TBX22* participated in the development of non-syndromic cleft palate and was reported to be associated with the risk of hypodontia (Braybrook et al., 2001; Kantaputra et al., 2011). To the best of our knowledge, this is the first research to provide new insights into dissecting the clinical value and potential biological roles of *TBX22* in PTC.

In the present study, we demonstrated that *TBX22* was significantly downregulated in PTC tissues compared with normal thyroid tissues in four public datasets, which contained more than 650 PTC patients. We then examined the *TBX22* mRNA level by our own RT-qPCR profiles as a validated cohort, including 79 matched PTC and adjacent normal tissues. PTC patients with high tumor stage or poor differentiation still have a certain rate of metastasis and mortality with the worse outcomes (Farahati et al., 2004). We also found that *TBX22* expression was negatively associated with advanced clinicopathological characteristics, including larger tumor size, LNM, higher disease stage, and extrathyroidal extension. It reflected that *TBX22* might be associated with the progression of PTC through TCGA analysis. As a validation, we found that *TBX22* was also associated with tumor size and LNM in our local cohort. Using the ROC analysis, *TBX22* level can perfectly predict PTC and normal thyroid tissue types in the four publicly available datasets and our local cohort (all AUC > 0.91). Besides, *TBX22* can also predict T stage and LNM status in the TCGA and local cohorts. All these results indicate that *TBX22* is a potential biomarker in PTC.

In addition to the traditional clinicopathological risk factors, specific molecular profiles (e.g., *BRAF*, *TERT*, and *RET*) may be used to predict the risk of extrathyroidal extension and lymph node metastases (Xing et al., 2005; Lee et al., 2007; Kim et al., 2012). The relative simplicity of the PTC genome with dominant mutually exclusive driving events enabled previous researches to clearly dissect the fundamental difference in genomic, epigenomic, and proteomic profiles between BVL and RL. In a previous multivariate analysis, the only clinicopathologically significant predictor of persistent disease after 5 years of follow-up was the presence of mutated *BRAF*^*V*600*E*^ (Elisei et al., 2012). We observed that the *TBX22* expression is much lower in *BRAF*^*V*600*E*^ mutated, *RAS* wild-type, and BVL phenotypes. *TBX22* can predict *BRAF*^*V*600*E*^ mutated status in the TCGA and local cohorts using ROC curves. *TERT* mutation had a significantly higher prevalence in aggressive thyroid cancer and served as an independent predictor of persistent disease and mortality for differentiated thyroid cancer. *RET* fusions commonly had BVL phenotype and upregulated MAPK activity. In this study, we found that *TBX22* expression was significantly lower in *TERT* mutation and *RET* wild-type groups. VEGF is a potent stimulator of endothelial cell proliferation, which may help predict the presence of metastases. In a retrospective study of 19 patients with PTC, Klein et al. (2001) had shown that a high level of VEGF correlated with a high risk of metastatic disease. RNA-seq profile from the database showed that *TBX22* had a strong positive correlation to VEGF expression. TMB is a novel emerging indicator of immunotherapy and reflects the total mutation index of tumor samples (Chalmers et al., 2017). Tumors with a higher mutation burden are hypothesized to have more opportunities to produce neoantigens that can be recognized by the immune system (Chan et al., 2019). MSI has been found in many diverse cancer types, including colorectal, endometrial, gastric, prostate, and thyroid cancer (Hause et al., 2016; Genutis et al., 2019). Previous studies showed that MSI-high tumors tend to be poorly differentiated and have an expansile growth pattern, histological heterogeneity, and increased tumor-infiltrating lymphocytes (Zheng et al., 2020). Interestingly, we observed a weak correlation between *TBX22* and the status of TMB and MSI in PTC samples. These features are well-known molecular characteristics that affect the prognosis of patients. However, although a number of molecular characteristics were identified to be associated with *TBX22* expression, we found that *TBX22* is not an independent prognostic biomarker in PTC. This may be due to the good prognosis of most PTC patients, leading to a small incidence of DFI and PFI events. These lines of evidence imply that *TBX22* may participate in the regulation of PTC tumorigenesis and progression.

To functionally interpret *TBX22* in the PTC, we investigated possible mechanisms by which *TBX22* affects PTC outcomes from transcriptomic aspects. Co-expressed genes usually act synergistically in biological processes under strict regulatory control, thus having an advantage in adaptive evolution (Niehrs and Pollet, 1999). TBX22^*low*^ status was associated with advanced clinical, pathological, and molecular features. *TBX22* expression was also associated with the enrichment of gene sets related to the MAPK pathway and thyroid hormone metabolism. More importantly, other than cancer signaling pathways, immune processes such as IL-2 and IL-6 signaling pathways, INF-α and INF-γ response, and complement activity were dominantly associated with TBX22^*low*^ status (14 terms were significant GSEA results, 13 were immune- or cancer-related processes). A deeper analysis of the complexity within TME may promote identifying patient populations for neoadjuvant treatment targets (Lin et al., 2019). Here, we explored the relationship between TME score with *TBX22* expression based on two different computational algorithms. Our study identified that *TBX22* expression was negatively associated with immune scores but not significant with stromal scores. Based on the functional enrichment analysis of *TBX22* and its correlation with TIME above, we hypothesized that *TBX22* was associated with immune cell component in the PTC microenvironment.

The deconvolution technique has been proven to be useful in the decomposition of heterogeneous cellular admixture of TME (Gentles et al., 2015; Orhan et al., 2020). As there is no gold standard for inferring immune infiltrate from the RNA-seq profile, we chose ssGSEA-based ImmuCellAI and deconvolution-based quanTIseq algorithms in inferring the composition of TIICs. Cunha et al. (2012, 2015) demonstrated that a higher CD8^+^ T-cell infiltration level was related to better disease-free survival rate and increased risk of relapse of PTC. A previous study indicated that tumors with high CD8^+^ T cells are more likely to respond to immunotherapy than the low CD8^+^ T-cell tumors (Farhood et al., 2019). Furthermore, an increase in anticancer CD8^+^ T, Tfh, and Th1 cells and M1 macrophages and a decrease in procancer M2 macrophages were associated with TBX22^*low*^ in PTC. In addition, we also found that the distribution of CD4^+^ T cells was accordantly lower in the TBX22^*low*^ group. We noticed that the infiltration levels of procancerous CD4^+^ T and Tregs cells in TBX22^*low*^ samples were higher, and procancerous Th2 cells had an elevated activity score in cancer immune cycle in TBX22^*low*^ samples, which may lead to the cancer-promoting phenotype corresponding to TBX22^*low*^ status (Hadrup et al., 2013; Tanaka and Sakaguchi, 2017; Wu et al., 2017).

The main goal of immunotherapy is to trigger the cancer immunity cycle without damaging normal cells. Based on this consideration, many current immunotherapeutic agents like CTLA-4 and PD-L1 inhibitors target steps in the cancer immunity cycle (Chen and Mellman, 2017). Patients who have lower *TBX22* expression showed higher anticancer immunity scores in different steps of the cancer immunity cycle. We found that lower *TBX22* expression led to more recruitment of anticancer CD8^+^ T cells, Th1, Tregs, and NK cells, which conform to the results of TIME alteration. Besides, we found that TBX22^*low*^ did appear to be associated with a favorable TIME in PTC. These results support that *TBX22* may manipulate the activity of the cancer immunity cycle.

TIICs in the TME may affect tumor cell proliferation, metastasis, and therapy resistance (Gocheva et al., 2010; Joyce and Fearon, 2015). However, there has been no experimental study to elucidate the functional effects of *TBX22* dysregulation in PTC models. Transfection efficiency and background expression of the *TBX22* in different PTC cell lines were considered. The significance of *TBX22* is demonstrated through loss-of-function, which increased the proliferation, migration, and invasion ability of PTC cells. Conversely, the gain-of-function addition of *TBX22* to PTC cells demonstrated attenuated proliferation, migration, and invasion. Together, these findings suggest that *TBX22* exerts a considerable tumor-suppressor effect in PTC.

The current study has several limitations. Firstly, more local tumor samples are needed to verify the relationship between *TBX22* and the clinical characteristics and confirm its predictive performance as a biomarker in PTC. Secondly, most of the existing TME computational methods are limited to only a single omics layer, which may not be enough to detect particular biological processes. It is necessary to quantify the particular biological processes and TME heterogeneity by multidimensional omics data. Thirdly, our research revealed a markedly close interaction between *TBX22* and anticancer immunity, as well as tumorigenesis and progression in PTC. However, the underlying *TBX22*-related mechanisms in PTC are yet to be fully elucidated. Constructing *in vivo* models to explore the potential biological mechanisms of *TBX22* is the direction of future research. Lastly, the potential cross talk between the *BRAF*^*V*600*E*^ mutation and TIME alteration in *TBX22*-induced PTC progression requires further exploration.

## Conclusion

The involvement of *TBX22* in PTC appears to be highly complex. Our study is the first to identify the expression level and the relationship with the clinicopathological and molecular features, tumor-infiltrating, cancer immunity cycle, and functional effects of *TBX22* in PTC. The present study sheds light to conduct further *TBX22*-associated research to assess its efficacy and receptiveness in PTC treatment.

## Data Availability Statement

The original contributions generated for this study are included in the article/Supplementary Material, further inquiries can be directed to the corresponding auth or/s.

## Ethics Statement

All research protocols have been approved and implemented through the ethical standards of the institutional review board of the First Affiliated Hospital of Wenzhou Medical University (Approval No. 2012-57). The patients/participants provided their written informed consent to participate in this study.

## Author Contributions

XD and JS contributed to study design, bioinformatic analysis, and manuscript draft. JH and CZ contributed to sample preparation and molecular biology experiments. XZ and HL contributed to the revision of the manuscript. All authors read and approved the final manuscript.

## Conflict of Interest

The authors declare that the research was conducted in the absence of any commercial or financial relationships that could be construed as a potential conflict of interest.

## Supplementary Material

Supplementary Table 1 Univariate Cox regression of clinical and pathological factors in PTC patients.

Supplementary Table 2 *TBX22* co-expressed genes detailed results.

Supplementary Table 3 GO enrichment detailed results.

Supplementary Table 4 KEGG enrichment detailed results.

Supplementary Table 5 GSEA enrichment detailed results.

## Abbreviations

PTC: papillary thyroid cancer
TBX22: T-box transcription factor 22
BVL: BRAF^*V600E*^-like
RL: RAS-like
CTLA-4: cytotoxic T lymphocyte-associated antigen 4
TME: tumor microenvironment
TIICs: tumor-infiltrating immune cells
TIME: tumor immune microenvironment
DFI: disease-free interval
PFI: progression-free interval
TMB: tumor mutation burden
MSI: microsatellite instability
GO: Gene Ontology
KEGG: Kyoto Encyclopedia of Genes and Genomes
GSEA: gene set enrichment analysis
TIP: tumor immunophenotype
MAPK: mitogen-activated protein kinase
ssGSEA: single-sample gene set enrichment analysis
PCR: polymerase chain reaction
NC: negative control
CCK-8: Cell Counting Kit-8
OD: optical density
ANOVA: analysis of variance
ROC: receiver-operating characteristic
LNM: lymph node metastasis
VEGFA: vascular endothelial growth factor A
AUC: area under the curve
RSEM: RNA-seq by expectation-maximization
Tc: cytotoxic T cells
Tem: central memory T cells
Tex: exhausted T cells
Tfh: follicular helper T cells
MAIT: mucosal-associated invariant T cells
NK: natural killer cells
MDSC: myeloid-derived suppressor cells.
